# Evaluating the effectiveness of a social and emotional learning program among preschool children in Japan: an experimental cohort study

**DOI:** 10.1186/s13034-023-00643-6

**Published:** 2023-08-03

**Authors:** Rikuya Hosokawa, Yuki Matsumoto, Chizuko Nishida, Keiko Funato, Aki Mitani

**Affiliations:** 1 Department of Human Health Sciences, Graduate School of Medicine, Kyoto University, 53 Kawara-Cho Shogo-in, Sakyo-Ku, Kyoto, 606-8507 Japan; 2 School of Human Life Sciences, Tokushima Bunri University, Tokushima, 770-8514 Japan; 3 Tanabe City Shinjyo Daini Elementary School, Wakayama, 646-0011 Japan; 4 Shirahama Town Shirahama Gakudo Nursery School, Wakayama, 649-2211 Japan; 5 Minabe Ainosono Childcare Center, Wakayama, 645-0005 Japan

**Keywords:** Social and emotional learning, Fun FRIENDS program, Preschool children, Japan

## Abstract

**Background:**

Research on school maladjustment has increasingly focused on social skills, such as the ability to control emotions, collaborate with others, and achieve goals. Social and emotional learning (SEL) is one approach to nurturing social skills. However, few preventive interventions to promote SEL have been conducted among young children, particularly in Asian countries, including Japan. Therefore, this study examined the effectiveness of an SEL program—Fun FRIENDS—among children in Japan.

**Methods:**

In mid-2022, the Fun FRIENDS program was administered to 115 children aged 4–5 years, who were enrolled in two kindergartens. The program was administered to the entire class as part of their kindergarten activities. The control group included 93 children in three kindergartens. This study included 94 participants (81.7%) in the intervention group and 66 (71.0%) in the control group, whose parents agreed with the assessment of their skills. Fun FRIENDS is a support program based on a cognitive–behavioral approach. The program aims to teach children how to cope with anxiety and stress and develop resilience and confidence to face difficulties. The program includes 10 sessions, each lasting approximately 1 h and conducted once per week. To examine the program’s effectiveness, teachers evaluated these children’s social skills before and after program implementation using the Social Skill Scale.

**Results:**

Results showed significant post-intervention improvements in self-control and cooperation scores among children in the intervention group, compared with pre-intervention. Further, post-intervention self-control and cooperation scores were significantly higher among children in the intervention group than the control group.

**Conclusions:**

SEL implemented on a class-wide basis could be effective in early childhood. An early approach targeting preschool-aged children is necessary to prevent school maladjustment. A universal approach implemented on a whole-class basis could contribute to improving children’s social skills.

**Supplementary Information:**

The online version contains supplementary material available at 10.1186/s13034-023-00643-6.

## Background

In recent years, school maladjustment has been increasing in Japan, with the number of truant students in elementary and junior high schools reaching 240000 in 2021, the highest number ever recorded in the country [1]. This increase has been accompanied by a growing interest in social skills among researchers and policymakers [1]. Social skills, also known as non-cognitive skills, include the ability to control emotions, collaborate, and achieve goals [2, 3]. Kindergartens and nurseries, where children spend a substantial part of their preschool years, are important environments for nurturing social skills; therefore, considerable attention is devoted to enhancing these skills in early childhood education and care [2, 3]. Appropriate support is necessary during the transition from infancy to school age, to prevent maladjustment in school life [2, 3]. Moreover, support methods to address school maladjustment, such as truancy, withdrawal, and behavioral problems, are being sought [2, 3]. Problems in continuity from early childhood to school age pose a higher risk for families with lower socioeconomic status and could be linked to the cycle of poverty [4, 5]. However, while the importance of preschool support has been established, the efforts that could effectively foster social skills are yet to be clarified [4, 5]. In other countries, preschool measures targeting families with low socioeconomic status in poor areas have been implemented, and the effectiveness of these strategies has been confirmed [6]. In Japan, similar measures have been limited. As families with low socioeconomic status are not necessarily concentrated in one area, and the relative poverty rate in Japan is characterized as high among Organisation for Economic Co-operation and Development countries [7], it is difficult to establish a general approach. Thus, to implement effective measures in Japan, adopting a universal approach that will not limit the target to high-risk families is crucial.

The concept of social and emotional learning (SEL) involves the development of social–emotional skills [8, 9]. The Collaborative for Academic, Social, and Emotional Learning (CASEL), a nonprofit organization based in the United States, was established in 1994 to define SEL [8, 9]. CASEL identified five competencies that children acquire through SEL: self-awareness, self-management, social awareness, relationship skills, and responsible decision-making [8, 9]. Several universal programs have been developed for use as part of SEL, and their effectiveness have been reported primarily in countries other than Japan [10–12]. FRIENDS, a support program based on a cognitive–behavioral approach, is one such program [13, 14].

FRIENDS programs are supported by the World Health Organization as evidence-based programs, and a meta-analytic review of the programs provides evidence [15]. FRIENDS is grounded in cognitive–behavioral therapy and is activity- and play-based [15]. Variations of the program have been adapted for all age groups—preschool through adulthood [15]. The purposes and practices of the program in schools and preschools fit well into the SEL framework [16]. It includes a series of programs: Fun FRIENDS for 4–7-year-olds, FRIENDS for life for 8–11-year-olds, My FRIENDS Youth for 12–15-year-olds, FRIENDS for children aged 16 years or older, and Adult Resilience for individuals aged 16 years or older [13, 14]. These programs were developed to meet the needs of children at each developmental stage [13, 14]. Moreover, their effectiveness has been verified in Australia, the United Kingdom, the United States, Canada, and Mexico, among others; and they have been reported to reduce anxiety and depressive symptoms, develop social and cognitive skills, and improve resilience and self-esteem [17]. Fun FRIENDS, which is designed for use with young children, aims to help them learn how to cope with anxiety and stress as well as develop resilience and confidence to face difficulties. However, its validation has been scarce, especially in Asian countries such as Japan [17].

This program was developed in Australia, and its effectiveness has been demonstrated in Western countries. The effectiveness of the program could differ between Western and Asian countries, where there are differences in linguistic and non-linguistic expressions. This study was conducted to verify whether the program developed in Australia would be effective in Japan. The ultimate goal is to reduce the poverty gap using SEL and preventing school maladjustment. Early preventive intervention during childhood is highly effective for improving and managing mental health not only during school age but also during adolescence and adulthood [18–20]. However, few SEL preventive interventions for young children have been conducted in Japan; although the effectiveness of Fun FRIENDS has been examined among elementary school children [21], the effectiveness of Fun FRIENDS has not been fully tested among young children in Japan. Consequently, to prevent school maladjustment, examining the effectiveness of Fun FRIENDS targeting young children is crucial. In kindergartens, Fun FRIENDS has been implemented as a preschool initiative. Therefore, this study measured this program’s effectiveness by assessing children’s social skills before and after the program. We hypothesized that children’s social skills would improve after program implementation.

## Methods

### Participants

**Fig. 1:**
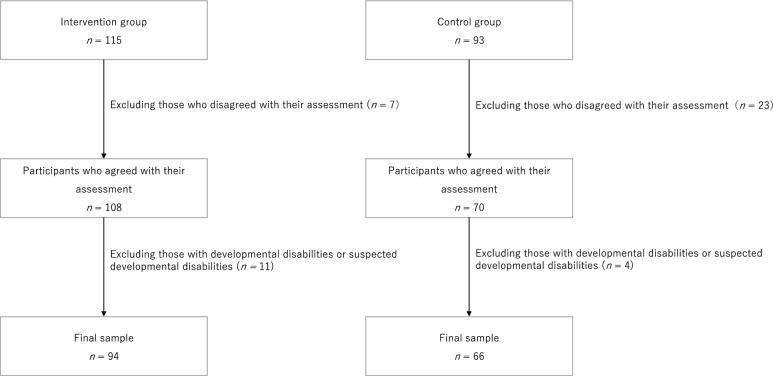
Study participants

In 2022, the Fun FRIENDS program, a major SEL program worldwide, was implemented for 4-year-old children enrolled in a middle-year kindergarten class in Wakayama Prefecture, a suburb in Japan. The intervention group comprised 115 participants from two kindergartens. The control group comprised 93 participants from three kindergartens. To ensure an adequate sample size, this study was conducted at several kindergartens. Kindergartens with similar educational policies were selected. Children whose parents disagreed with the assessment of their skills (intervention group: *n* = 7, control group: *n* = 23) were excluded from the statistical analysis but were still offered the intervention program. Children with language disorders or pervasive developmental disorders (intervention group: *n* = 11, control group: *n* = 4) were excluded from the statistical analysis; however, they were also offered the intervention program. Such disorders were assessed based on various parent and teacher reports. Consequently, the analysis included 94 children in the intervention group and 66 children in the control group (Fig. 1).

### Procedure

Teachers were asked to administer the Social Skills Questionnaire for Preschoolers (SSQ-P) before and immediately after the intervention. The Fun FRIENDS program was implemented by facilitators who were trained teachers and have completed the educational course prescribed by Fun FRIENDS. The program was implemented over approximately 2.5 months. All sessions were attended by homeroom teachers.

### Intervention

The Fun FRIENDS program is designed to help children learn various ways to cope with anxiety and stress and to help them develop social skills, resilience and confidence in their ability to face difficulties [13, 14, 22]. The program includes 10 sessions lasting approximately one hour each, which are conducted once per week. The Fun FRIENDS program teaches children cognitive–behavioral strategies to address several areas of social–emotional learning. The program utilizes play-based activities to deliver skills in a developmentally appropriate manner. The program name, Fun FRIENDS, is an acronym for the strategies taught in the program; each letter corresponds to a component of the program. Thus, FRIENDS is an acronym for the theme of each session: F-Feelings, R-Relax, I-I can try, E-Encourage, N-Nurture, and D-Do not forget to be brave! For a detailed description of the contents of the sessions, see Additional file 1: Appendix S1 [22], as well as Pahl and Barrett [13]. A facilitator with expertise in the relevant field conducted the program. A Japanese version of the materials was prepared, and several facilitators were involved in the program to ensure its quality.

In 2008, a pilot study was conducted with Japanese families living in Brisbane, Australia. The founder, Dr. Barrett, provided resources to Dr. Matsumoto, who led the research and translated the manual and workbook into Japanese. Cultural adaptations or considerations, such as characters, activities, and time frames, were discussed between them. Additionally, the program has been implemented with preschoolers in child care settings in Japan since 2010.

## Measures

### Outcome variables

Social skills were measured using the teacher-reported SSQ-P [23]. The SSQ-P was developed in Japan and comprises three measures of children’s social skills. It contains 24 items divided into three subscales (i.e., assertion, self-control, and cooperation) [24, 25]. These subscales are based on the Social Skills Rating System (SSRS) introduced by Gresham and Elliott [26] and are positively correlated with social skills of the SSRS. This rating system is one of the most widely used social skill scales and has been used in influential National Institute of Child Health and Human Development studies [27, 28]. Items are rated on a three-point Likert scale (0 = *never*, 1 = *sometimes*, and 2 = *very often*), with higher scores indicating better social skills. The utilized subscale has shown sufficient internal consistency, construct validity, and internal reliability in previous studies. In addition, the researcher fully explained the SSQ-P to the teachers to ensure that they could use it accurately.

### Explanatory variable

The presence or absence of the Fun FRIENDS program intervention (intervention and control groups) was the explanatory variable.

### Demographics

Parents reported information on their family structure (single-parent or two-parent family), household income (< 4 million Japanese yen [JPY], ≥ 4–8 million JPY, ≥ 8 million JPY), and parents’ educational level (middle school/high school, junior college/vocational school, university, or graduate school).

### Statistical analyses

A chi-squared test was used to examine differences in attributes between the intervention and control groups at baseline. We also analyzed the differences between the intervention and control groups before and after program implementation using an independent *t*-test. Further, we analyzed changes in social skills before and after the program (T1 vs. T2) in the intervention and control groups using paired-samples *t*-tests. All analyses were conducted using SPSS 29.0 (IBM, Armonk, NY, USA).

### Ethics statement

As Fun FRIENDS was a preschool initiative, all children aged 4–5 years enrolled in the included kindergartens participated in the program. Prior to the intervention, the parents were informed of the study purpose and associated procedures and were made aware that participation was voluntary. Parents provided written informed consent for themselves as well as on behalf of their children before participation. This study was approved by the Kyoto University Ethics Committee (C1563).

## Results

### Study participant demographics at T1

Table 1 shows participants’ demographics at T1. The average age of the children was 4.72 (± 0.33) years for the intervention group and 4.82 (± 0.33) years for the control group. Participants’ sex distribution was 51 (54.26%) and 30 boys (45.45%) in the intervention and control groups, respectively. The differences in attributes between the intervention and control groups were assessed using a chi-squared test. No significant differences were found.

**Table 1:** Participant characteristics at T1

	*N*	Intervention group (*n* = 94)		Control group (*n* = 66)		*p*
		*n*	%	*n*	%	
Child’s sex						
Male	81	51	62.96	30	37.04	0.273
Female	79	43	54.43	36	45.57	
Family composition						
Single-parent family	9	3	33.33	6	66.67	0.111
Two-parent family	151	91	60.26	60	39.74	
Presence of siblings						
0	32	18	56.3	14	43.8	0.748
≥ 1	128	76	59.4	52	40.6	
Annual household income (million JPY)						
< 4	31	17	54.84	14	45.16	0.796
≥ 4–8	80	49	61.25	31	38.75	
≥ 8	30	17	56.67	13	43.33	
Maternal educational level						
Middle school or high school	60	36	60.00	24	40.00	0.167
Junior college or vocational school	55	34	61.82	21	38.18	
University or graduate school	37	16	43.24	21	56.76	
Paternal educational level						
Middle school or high school	53	32	60.38	21	39.62	0.913
Junior college or vocational school	31	18	58.06	13	41.94	
University or graduate school	62	35	56.45	27	43.55	

T1, before program implementation (baseline); *JPY* Japanese yen. The median annual household income in Japan was approximately 4 million yen as of 2021 [29]

### Differences in social skills (intervention vs. control group)

**Fig. 2:**
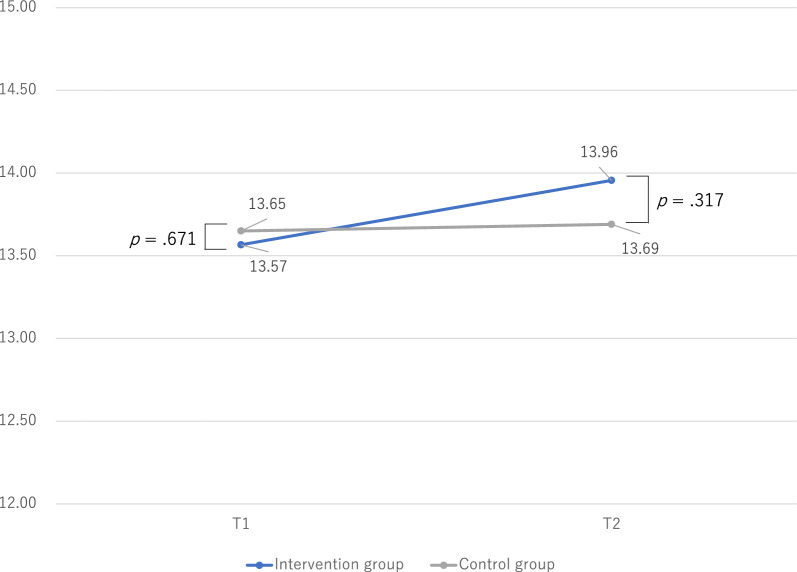
Changes in social skills: assertion

**Fig. 3:**
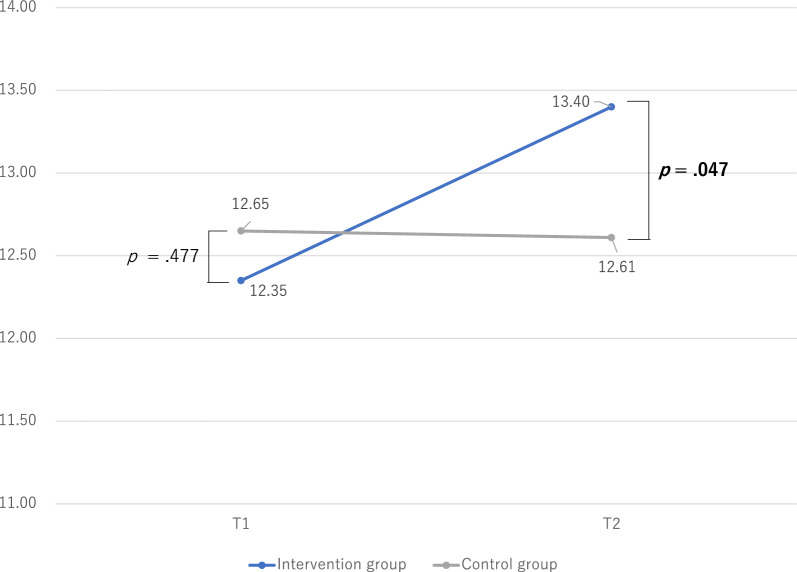
Changes in social skills: self-control

**Fig. 4:**
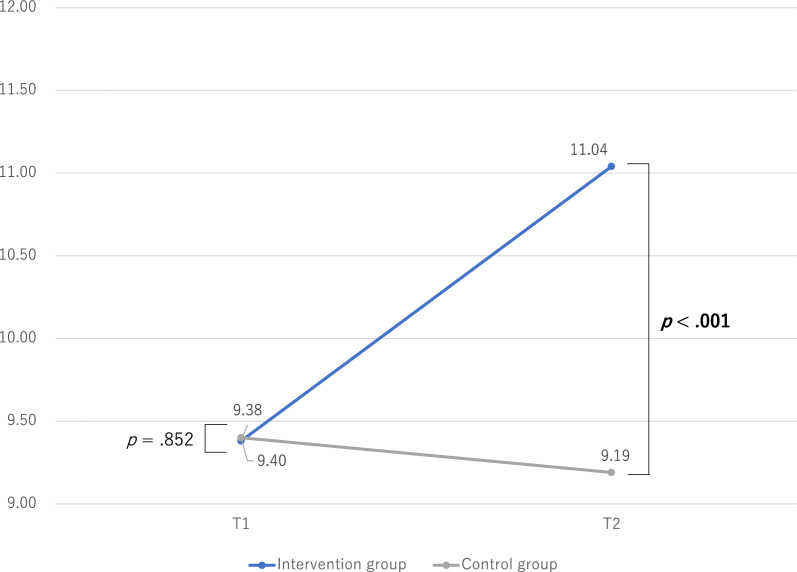
Changes in social skills: cooperation

Figures 2, 3 and 4 show the differences in changes in social skills between the intervention and control groups. The differences in social skills in the intervention and control groups were examined using an independent *t*-test. There were no significant differences in social skills between the intervention and control groups at T1. However, the intervention group had significantly higher self-control and cooperation scores than the control group at T2.

### Changes in social skills (T1 vs. T2)

Table 2 shows the changes in social skills (T1 vs. T2). The differences in social skills between T1 and T2 were examined using paired-samples *t-*tests. In the intervention group, social skills were significantly higher in self-control and cooperation at T2 than at T1. However, the control group did not exhibit significant differences in social skills between T1 and T2.

**Table 2:** Changes in social skills (T1 vs. T2)

	Intervention group			Control group		
	M	SD	p	M	SD	p
Assertion						
T1	13.57	2.72	0.133	13.65	2.43	0.710
T2	13.92	2.81		13.69	2.84	
Self-control						
T1	12.35	3.31	**0.044**	12.65	3.65	0.874
T2	13.40	3.49		12.61	3.51	
Cooperation						
T1	9.38	5.98	** <0 .001**	9.40	4.14	0.778
T2	11.04	5.80		9.19	5.99	

T1, before program implementation (baseline); T2, after program implementation; *M* median, *SD* standard deviation

Bold numbers indicate statistically significant differences

## Discussion

This study evaluated the effectiveness of Fun FRIENDS, a universal prevention intervention program, among preschool children. Compared with the control group, the intervention group showed significantly greater increase in self-control and cooperation after the intervention.

Children’s self-control and cooperation improved after the intervention. These social competencies influence social adjustment during childhood and throughout life [30–33]. The Fun FRIENDS program teaches children various cognitive–behavioral strategies for addressing several areas of social–emotional learning [13, 14, 22]. The program is unique because, in addition to focusing on reducing negative aspects, it also promotes certain protective factors, such as resilience and wellbeing. This study utilized play-based activities to deliver skills in a developmentally appropriate manner to help children develop self-control. The program enhances self-control and cooperation through teaching, including understanding emotions of oneself and others, learning how to control emotions such as anger, and recognizing the presence of people one can rely on. The program contents included understanding and controlling one’s own emotions (see Additional file 1: Appendix S1 for details).

The results demonstrated the effectiveness of the Fun FRIENDS program for Japanese children. This program is structured based on cognitive–behavioral therapy. As such, it could be effective across cultural backgrounds. In addition, this could be because the kindergarten teachers also participated in the program, understood its content, and implemented the program’s learning outside of the program sessions. Prior research focused on the potential effectiveness of the Fun FRIENDS program as a universal school-based prevention intervention—that is, the program was offered to young children regardless of their risk status [34, 35]. As children attending preschool spend much of their day in kindergarten, this environment provides a common entry point for providing interventions to several children. The kindergarten environment is a suitable pathway for identifying children who need support and for providing the services they are entitled to. Kindergartens are ideal places for implementing preventive intervention programs aimed at promoting social–emotional competence during early childhood. A universal approach is one that can be applied to the whole group or classroom. The Fun FRIENDS program teaches children cognitive–behavioral strategies to address several areas of social–emotional learning [13, 14, 22]. The program facilitates appropriate skill development utilizing play-based activities. Universal interventions are generally designed to promote general mental health or to build wellbeing and resilience.

Assertion was not affected by this program. This program teaches children a variety of cognitive–behavioral strategies to address several areas of social–emotional learning. The program utilizes play-based activities and provided skills in a developmentally appropriate manner to help children develop cooperation and self-control. The program enhances self-control and cooperation through teaching, including understanding one’s own and others’ emotions, learning how to control emotions such as anger, and recognizing the presence of people one can rely on. Therefore, contents contributing to assertion may have been scarce.

### Limitations and future research directions

Despite this study’s strengths and unique contributions, some limitations and future research directions must be noted. The greatest limitation of this analysis was that it was based solely on teachers’ reports of children’s social skills. Children’s subjective evaluations and evaluators’ objective evaluations should be added in future studies. Additionally, participants in the control group were not offered an alternative to the FRIENDS program. As the teachers in the control group were aware that the children in their classes were in the control group, a potential placebo effect could have occurred. Further, this analysis only evaluated the program immediately after its implementation. Neil and Christensen noted that without a long-term follow-up, potential effects could be missed, leading to an underestimation of the program’s effectiveness [36]. Further, the effectiveness of Fun FRIENDS has been tested. Antich reported that the intervention group had higher social–emotional skills than the control group, not only immediately after but also 12 months after the program [35]. Similar results were observed in a 12-month follow-up evaluation of the FRIENDS program for youth, implemented as a universal school-based intervention. Although conclusions about the program’s effectiveness in a 12-month follow-up were limited by the small sample size, available data showed that the effects were maintained during this period [37, 38]. Thus, ongoing testing is necessary to determine whether any long-term effects can be observed in childhood interventions. Further, the evaluation of social skills in this study relied on teacher reports. Although practical constraints make it difficult to include other treatment outcome measures in a community mental health setting, future studies should include other strategies for assessing outcomes (e.g., child-reported or third-party observation reports). In addition, as this study was conducted in Wakayama Prefecture, which is a rural region, the results may not be generalizable to other areas. Future research should include urban settings. Finally, the current intervention primarily targeted children. In addition to preschool programs, full-fledged interventions with parents are necessary to ensure the implementation of the relevant practices at home.

## Conclusions

Despite its limitations, the current results provide promising directions for future preventive interventions among young children. This study evaluated the effectiveness of Fun FRIENDS, a universal preventive intervention program, in preschool children. We hypothesized an increase in the children’s social skills post-intervention. Consistently, self-control and cooperation improved from pre- to post-intervention in the intervention group but not in the control group. This program is a unique child-centered intervention. Although further research is needed, this study indicated that direct interventions with children could be an effective strategy to improve social skills. Additionally, as this study was conducted in a general early childhood educational setting, the results can be used to assist future dissemination of the program.

### Supplementary Information

**Additional file 1: Appendix S1**. Outline of the Fun FRIENDS Session Content.

## Data Availability

The datasets generated and/or analyzed during this study are available from the corresponding author upon reasonable request.

## Abbreviations

CASEL: The collaborative for social and emotional learning
FRIENDS: F-Feelings, R-Relax, I-I can try, E-Encourage, N-Nurture, and D-Do not forget to be brave!
JPY: Japanese yen
SEL: Social and emotional learning
SSQ-P: Social Skills Questionnaire for Preschoolers
SSRS: Social Skills Rating System
