# Supplementary material for: Evaluating the effectiveness of a social and emotional learning program among preschool children in Japan: an experimental cohort study
Source: Child Adolesc Psychiatry Ment Health. 2023 Aug 3;17:93. doi: 10.1186/s13034-023-00643-6 (PMC10401874; doi:10.1186/s13034-023-00643-6)
Supplement: Supplementary file 1 — Additional file 1: Appendix S1. Outline of the Fun FRIENDS Session Content. [file 13034_2023_643_MOESM1_ESM.docx]

Appendix. Outline of the Fun FRIENDS Session Content

| Session | Session content: major learning objectives |
| --- | --- |
| 1 | - Developing a sense of identity; introduction to the group; name games. - Introduction to the concept of “being brave;” social skills promotion. - Acceptance of differences. |
| 2 | F: Feelings   - Affective education as well as identification of various emotions (e.g., recognition of physiological arousal associated with emotions). |
| 3 | F: Feelings (continued)   - How to cope with feelings, thumbs-up ideas (helpful coping behaviors), and thumbs-down ideas (unhelpful coping behaviors). - Building an understanding of the link between feelings and behavior. - Strategies for helping others when they experience negative feelings; this assists with the development of empathy. |
| 4 | R: Remember to relax   - Identification of physiological arousal (“body clues”) related to anxiety. - Certain relaxation strategies are taught (e.g., diaphragmatic breathing [milkshake breathing], progressive muscle relaxation, and visualization). |
| 5 | I: I can try my best!   - Introduction to the cognitive components of the program. - Children are taught to become aware of and pay attention to their inner thoughts or self-talk. - Self-talk is referred to in terms of “red” (unhelpful thoughts) and “green” (helpful thoughts) based on a traffic light analogy. |
| 6 | I: I can try my best! (continued)   - Introduction to challenging unhelpful red thoughts and formulating alternative helpful green thoughts. - Applying green thoughts to achieve goals. |
| 7 | E: Encourage   - Trying new things by breaking tasks down into small steps and using green thoughts to help achieve goals. |
| 8 | N: Nurture   - Introduction to the idea of role models and support teams—that is, people who help us achieve our goals. |
| 9 | D: Do not forget to be brave   - Supporting teams continued. - Planning ahead for difficult situations. |
| 10 | S: Stay smiling   - Children celebrating their success after program completion. |
